# Context-Dependent Epithelial and Immune Programs Shape Intestinal Resilience or Vulnerability Following Prior Colitis

**DOI:** 10.1016/j.jcmgh.2026.101826

**Published:** 2026-06-10

**Authors:** Priyanka Biswas, Vishwas Mishra, Julia Sanchez-Garrido, Gad Frankel

**Affiliations:** Department of Life Sciences, Imperial College London, London, United Kingdom

**Keywords:** *C rodentium*, DSS Colitis, Enteropathogenic *Escherichia coli*, Epithelial Barrier Integrity, IL17A, Inflammatory Memory, Mucosal Immunity, Type III Secretion System Effectors

## Abstract

**Background & Aims:**

Prior intestinal inflammation can leave durable immune and epithelial alterations, yet how these changes influence responses to subsequent injury remains unclear. Infectious and sterile colitis share core features, including barrier disruption and cytokine secretion. We therefore investigated whether the nature of the initial inflammatory event shapes protection or susceptibility during later intestinal insult.

**Methods:**

We used reciprocal mouse models of *Citrobacter rodentium* infection and dextran sodium sulphate–induced colitis to define how prior infectious vs sterile colitis shapes secondary disease. Barrier integrity, immune cell populations, cytokine production, and susceptibility to wild-type and *C rodentium* mutants that cause limited epithelial barrier disruption were assessed.

**Results:**

Mice recovered from *C rodentium* infection were protected against dextran sodium sulphate–induced colitis, displaying reduced weight loss, preserved epithelial architecture, and lower inflammatory pathology. This protection required type III secretion system effector–mediated epithelial injury during primary infection and was associated with sustained interleukin17A signaling, which contributed to the protective phenotype. In contrast, mice recovered from dextran sodium sulphate–induced colitis exhibited persistent epithelial barrier defects, chronic colonic neutrophilia, and heightened susceptibility to *C rodentium* infection despite elevated interleukin17A. Infection with *C rodentium* mutants that cause minimal epithelial damage still resulted in severe disease in dextran sodium sulphate–experienced mice, indicating that unresolved epithelial barrier dysfunction is a major contributor to vulnerability.

**Conclusions:**

The nature of the primary colitis is associated with distinct epithelial and immune programs that persist beyond resolution of inflammation. Infectious colitis is associated with a protective mucosal state where interleukin17A is a key contributor in a broader protective response, whereas sterile colitis is associated with persistent epithelial barrier dysfunction that is associated with increased susceptibility to subsequent infection. These findings highlight how inflammatory history influences long-term intestinal resilience or vulnerability.

## Graphical abstract

**Figure:**
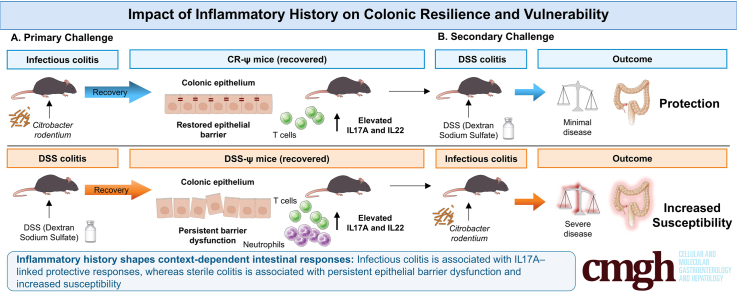

**Summary**

The authors show that prior infectious or chemically induced colitis differentially influences responses to subsequent intestinal insults, with interleukin17A contributing to protection and persistent epithelial barrier dysfunction associated with increased susceptibility, respectively.

**What You Need to Know**

**Background**

Prior intestinal inflammation induces lasting immune and epithelial changes, but how these adaptations influence responses to subsequent injury remains unclear despite shared pathologic features across infectious and sterile colitis.

**Impact**

Primary colitis establishes distinct epithelial and immune states, where infectious colitis promotes interleukin17A–associated protection, whereas sterile colitis drives persistent barrier dysfunction and increased susceptibility to subsequent infection.

**Future Directions**

Future studies will define molecular mechanisms driving persistent epithelial and immune alterations and determine how these changes influence responses to diverse inflammatory insults locally and systemically.

Intestinal mucosal injury can arise from diverse triggers, including enteric infection and chronic inflammatory disorders such as inflammatory bowel disease (IBD).^1^ Although their initiating causes differ, both infectious and noninfectious colitis converge on common pathologic features including epithelial barrier disruption, cytokine-driven inflammation, and immune cell infiltration.^1^^,^^2^ These shared mechanisms raise the possibility that the immune and epithelial adaptations established during 1 form of intestinal inflammation may influence how the host responds to future mucosal insults. Tissue-resident lymphocytes and innate lymphoid cells retain memory of prior inflammation,^3^^,^^4^ but whether such mucosal memory influences future pathology remains largely unexplored.

*Citrobacter rodentium* (CR), an extracellular attaching and effacing pathogen that models human enteropathogenic and enterohemorrhagic *E coli* infections, has been instrumental in elucidating host–pathogen interactions at the intestinal interface.^2^^,^^5^ CR infects colonic epithelial cells using a type III secretion system that delivers a coordinated set of effector proteins acting in an interdependent network to modulate host signaling.^6^^,^^7^ These interactions drive colonic crypt hyperplasia (CCH), disrupt tight junctions, and compromise the barrier integrity. In resistant mice (eg, C57BL/6), CR infection induces robust innate and adaptive immune responses, leading to pathogen clearance and restoration of homeostasis.^2^^,^^5^ During CR infection, interleukin (IL)17A and IL22 secreted from group 3 innate lymphoid cells (ILC3s) and T helper 17 (Th17) cells act on epithelial cells to reinforce barrier integrity, limit bacterial dissemination, and promote repair.^8^^,^^9^ These responses leave a lasting imprint on the mucosal landscape.

In C57BL/6 mice, CR infection follows 5 defined phases. During the establishment phase (1–3 days postinfection [dpi]), bacteria colonize the cecal lymphoid patch.^2^^,^^5^^,^^8^ The expansion phase (4–8 dpi) is marked by rapid bacterial proliferation, intimate CR adherence to intestinal epithelial cells (IECs), disruption of tight junctions,^2^^,^^5^ and activation of both group 2 innate lymphoid cells and ILC3s.^8^^,^^10^^,^^11^ This phase is also characterized by the emergence of trained ILC3s, which persist for months, providing protection against reinfections.^10^ In the steady-state phase (8–12 dpi), high levels of bacterial shedding reflect stable colonization of the distal colon. The clearance phase (13–18 dpi) is characterized by IgG-mediated elimination of the pathogen and restoration of epithelial barrier integrity.^5^ Finally, the post clearance phase is characterized by lasting interferon γ (IFNγ) responses and tissue-resident memory CD4^+^ T cells.^12^, ^13^, ^14^

Similar to infectious colitis, sterile epithelial injury may likewise impose long-term remodeling of the mucosal environment and modify host susceptibility to subsequent infection. Dextran sodium sulphate (DSS)-induced colitis is a well-established model of ulcerative colitis that causes direct chemical injury to colonic epithelial cells, resulting in barrier breakdown, microbial translocation, and activation of innate and adaptive immunity.^15^^,^^16^ Although the mucosa appears to recover macroscopically after DSS withdrawal, sustained epithelial and immune alterations have been shown to last for several weeks post-treatment, including persistent proinflammatory cytokine expression and incomplete tight-junction repair.^17^ Although mice recovered from 1 episode of DSS-induced colitis display resistance to subsequent DSS administration,^13^ they have been shown to be susceptible to *Clostridioides difficile* infection.^18^ Such findings suggest that an episode of colitis can leave a long-lasting immune imprint that can influence the outcomes to subsequent enteric challenges, although this remains largely unexplored.

Both infection- and chemical-induced colitis engage overlapping cytokine networks centered around IL22 and IL17A. IL22 promotes secretion of antimicrobial peptides and epithelial regeneration and barrier repair,^19^^,^^20^ whereas IL17A plays context-dependent roles, acting as either protective or pathogenic depending on the inflammatory milieu.^21^^,^^22^ Defining how these cytokines integrate with epithelial repair pathways following distinct inflammatory contexts is critical for understanding long-term mucosal adaptation.

In this study, we established 2 reciprocal models to dissect how primary intestinal inflammation influences future disease susceptibilities. We demonstrate that a resolved CR infection confers durable protection against DSS-induced colitis, whereas recovery from DSS-induced colitis renders mice highly susceptible to subsequent CR infection. Together, these reciprocal models reveal how distinct inflammatory histories shape long-term epithelial–immune crosstalk, offering insight into the balance between mucosal protection and pathology.

## Results

### A History of *Citrobacter rodentium* Infection Protects Mice From a Secondary Chemical-Induced Colitis

**Figure 1:**
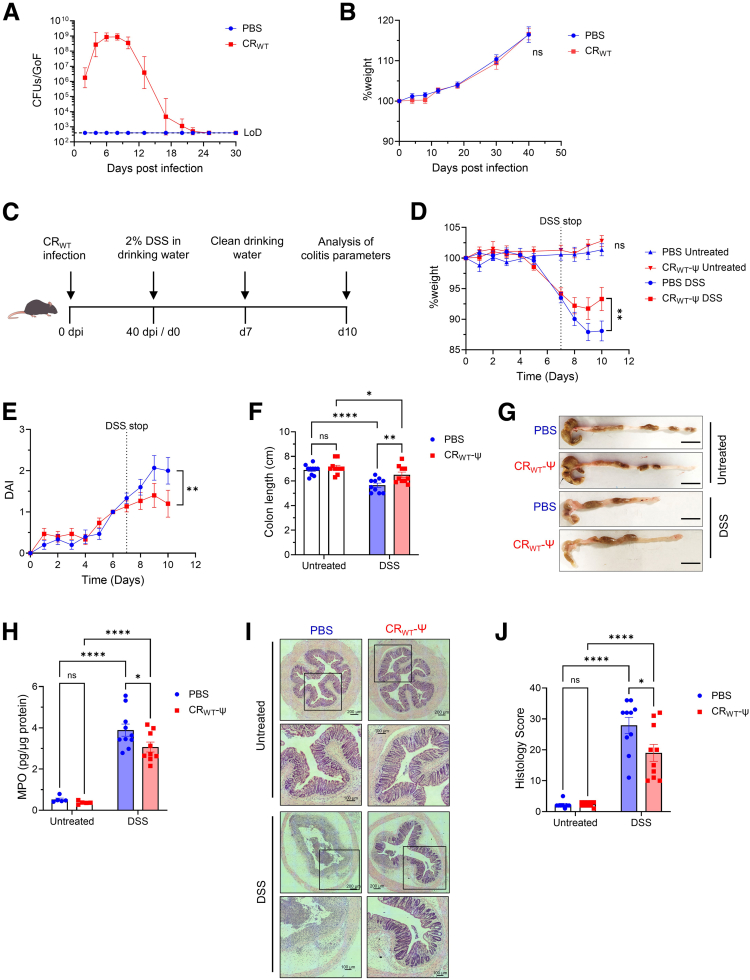
**A history of CR infection protects mice from DSS-induced colitis.** (*A*) Temporal fecal bacterial shedding following CR_WT_ infection. (*B*) Body weight change of mice postinfection. (*C*) Schematic representation of the experimental design: C57BL/6 mice were infected with CR_WT_ or mock-treated with PBS, followed by 2% DSS in drinking water at 40 dpi (CR_WT_-Ψ) for 7 days, with 3 days of clean water. (*D*) Temporal weight loss, (*E*) DAI, (*F*) colon length post-DSS treatment, (*G*) representative colon images, (*H*) colonic MPO levels, (*I*) representative hematoxylin and eosin (H&E)-stained colon sections post DSS treatment, and (*J*) histologic scores for H&E-stained colon sections. Scale, 200 μm. Each *dot* represents mean (*A, B, D*, and *E*) or individual mouse (*F, H*, and *J*). *Filled bars* represent data postsecondary challenge. Data shown are pooled values from a minimum of 3 biological repeats with 3 to 5 mice per group. Refer to Supplementary Table 1 for the exact number of mice used for experiments. Data shown are geometric mean ± standard deviation for (*A*). *P* values were determined on data plotted as mean ± SEM using 2-way analysis of variance with Sidak’s post-test for multiple comparisons. ns, not significant; ∗*P* < .05; ∗∗*P* < .01; ∗∗∗∗*P* < .0001.

To determine if a history of intestinal infection influences the outcome and susceptibility to colitis, we orally infected C57BL/6 mice with 10^9^ colony forming units (CFUs) of wild-type (WT) CR (CR_WT_); control mice received sterile phosphate-buffered saline (PBS). Temporal fecal shedding quantification revealed that CR was cleared by 21 dpi, and mice gained weight over time (Figure 1*A* and *B*). At 40 dpi (ie, ∼3 weeks post clearance) mice were treated with 2% DSS in drinking water for 7 days, followed by 3 days of clean drinking water (7+3 model) (Figure 1*C*). DSS treatment of control mice resulted in disease, as evidenced by weight loss from day 5 onwards (Figure 1*D*). In contrast, mice with a history of CR infection (CR_WT_-Ψ, the symbol ‘Ψ’ represents memory/history, hereafter) showed significantly higher weight gain and lower Disease Activity Index (DAI) post DSS withdrawal than mice with no history of infection (Figure 1*D* and *E*).

Colonic shortening and myeloperoxidase (MPO) are markers for colitis severity in mice.^15^^,^^23^ Similar colon length and colonic MPO were recorded in untreated PBS and CR_WT_-Ψ mice (Figure 1*F–H*). However, although significant shortening of colon was observed in both treatment groups of mice post DSS administration, a significantly longer colon was seen in CR_WT_-Ψ mice compared with PBS mice (Figure 1*F* and *G*). Moreover, the levels of colonic MPO were markedly reduced in CR_WT_-Ψ mice compared with PBS mice (Figure 1*H*).

Histologic analysis of distal colon revealed no discernible differences between untreated CR_WT_-Ψ mice and PBS mice, suggesting colonic recovery at 40 dpi (Figure 1*I* and *J*). DSS treatment resulted in colonic inflammation as marked by loss of colonic crypt architecture, immune cell infiltration, submucosal thickening, and epithelial disruption; however, CR_WT_-Ψ mice displayed lower extent of damage in comparison to PBS mice (Figure 1*I* and *J*). Together, these results demonstrate that, at 40 dpi, the colon shows recovery from the primary CR_WT_–mediated damage and inflammation; moreover, compared with the controls, mice with a history of CR_WT_ infection are protected from subsequent chemical-induced colitis.

**Figure 2:**
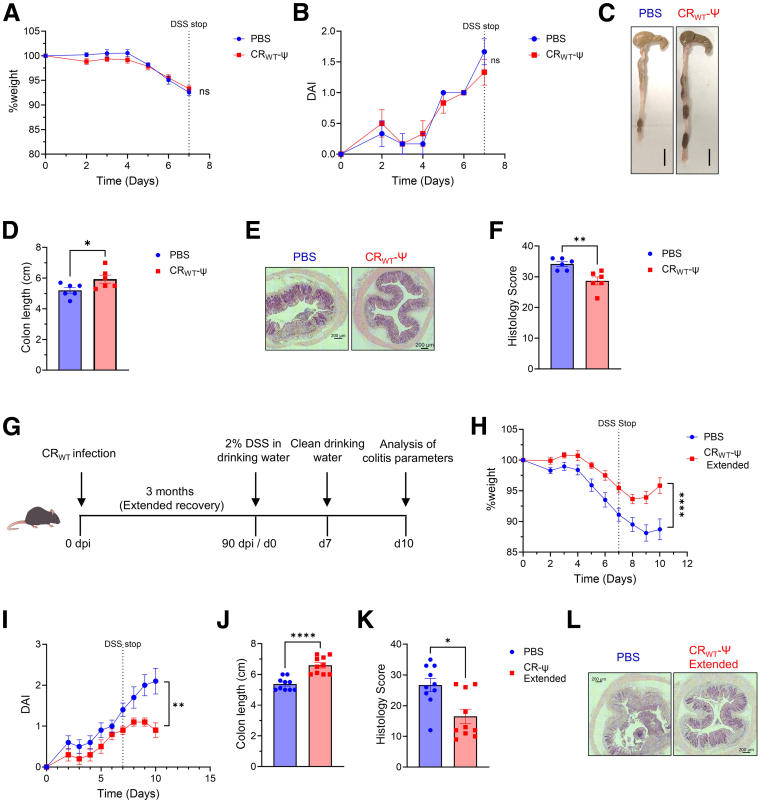
**Mice with a history of CR infection display sustained protection from DSS-induced colitis.** (*A–F*) Assessment of DSS-induced colitis at day 7 post DSS treatment in CR_WT_-Ψ and control mice. (*A*) Weight changes and (*B*) DAI during DSS treatment in PBS and CR_WT_-Ψ mice. (*C*) Representative colon images, (*D*) colon length, (*E*) representative hematoxylin and eosin (H&E)-stained colon sections, and (*F*) histologic scores for H&E-stained colon sections. Scale, 200 μm. (*G–L*) Assessment of DSS-induced colitis following extended recovery after CR infection (CR_WT_-Ψ Extended). (*G*) Schematic representation of the experimental design: C57BL/6 mice were infected with CR_WT_ or mock-treated with PBS, followed by 2% DSS in drinking water at 90 dpi for 7 days, with 3 days of clean water. (*H*) Weight loss and (*I*) DAI post-DSS treatment. (*J*) Colon length, (*K*) histologic scores, and (*L*) representative H&E-stained colon sections. Scale, 200 μm. Each *dot* represents mean (*A, B, H*, and *I*) or individual mouse (*D, F, J*, and *K*). Data shown are pooled values from a minimum of 2 biological repeats with 3 to 5 mice per group. Refer to Supplementary Table 1 for the exact number of mice used for experiments. *P* values were determined on data plotted as mean ± standard error of the mean using 2-way analysis of variance (*A, B, H*, and *I*), 2-tailed unpaired *t* test (*D* and *J*) and Mann–Whitney test (*F* and *K*). ns, not significant; ∗*P* < .05; ∗∗*P* < .01; ∗∗∗∗*P* < .0001.

Because PBS mice and CR_WT_-Ψ mice displayed similar kinetics of weight loss during the first 7 days of DSS administration, and the differences in disease were observed on day 10, we asked whether the differences observed were a consequence of lower disease during the DSS treatment or a better recovery after stopping DSS administration. To answer this, we sacrificed PBS mice and CR_WT_-Ψ mice on day 7 of DSS treatment. As observed earlier, PBS mice and CR_WT_-Ψ mice showed similar rate of weight loss (Figure 2*A*). Although CR_WT_-Ψ mice showed similar DAI compared with PBS mice (Figure 2*B*), they displayed lower colon shortening and colonic epithelial damage and inflammation as assessed in hematoxylin and eosin (H&E)-stained thin colon sections (Figure 2*C–F*). This suggests that CR_WT_-Ψ mice can tolerate DSS administration better than the control mice.

We assessed long-term protection capability by treating mice with 2% DSS, at 90 days post CR_WT_ infection, using PBS-gavaged mice as control (Figure 2*G*). CR_WT_-Ψ mice with extended recovery time showed better tolerance to DSS, as shown by lower weight loss and quicker weight gain than PBS mice (Figure 2*H*). CR_WT_-Ψ mice also displayed lower DAI (Figure 2*I*), as well as significantly longer colon length (Figure 2*J*). Histologic analysis of distal colonic sections revealed lower inflammation and epithelial damage in CR_WT_-Ψ mice as compared with PBS controls (Figure 2*K* and *L*). Together these results indicate that a history of CR_WT_ infection results in long-term protection against subsequent chemical-induced colitis.

### Mice Recovered From Dextran Sodium Sulfate–Induced Colitis Show Heightened Susceptibility to *Citrobacter rodentium* Infection

**Figure 3:**
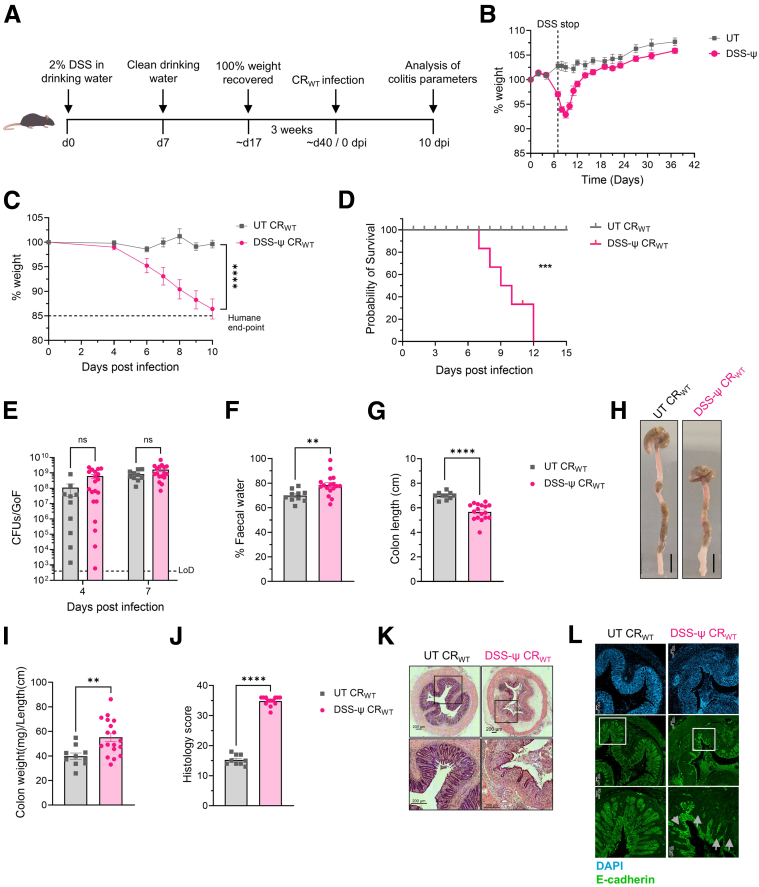
**Mice with a history of DSS are susceptible to subsequent CR infection.** (*A*) Schematic representation of the experimental design: C57BL/6 mice were treated with 2% DSS in drinking water, and 3 weeks post weight recovery were infected with CR_WT_ (DSS-ψ CR_WT_). DSS-untreated mice, infected with CR_WT_ (UT CR_WT_) were used as control. (*B*) Percentage weight change post DSS treatment and (*C*) CR_WT_ infection. (*D*) Probability of survival. (*E*) Temporal fecal bacterial shedding. (*F*) Fecal water content at 7 dpi. (*G*) Colon length, (*H*) representative colon images, (*I*) colon weight-to-length ratio, (*J*) histologic scores for hematoxylin and eosin (H&E)-stained colon sections, and (*K*) representative H&E-stained distal colon sections of mice harvested at 10 dpi. Scale, 200 μm. (*L*) Representative immunostaining images of colonic sections from CR_WT_-infected UT and DSS-ψ mice at 10 dpi. The IECs express E-cadherin (*green*) and are 4',6-diamidino-2-phenylindole (DAPI^+^; *blue*). In the zoomed E-cadherin panel, *gray arrows* indicate erosion of colonic epithelial layer, and loss of colonic crypt architecture represented by lower/loss of E-cadherin staining in DSS-ψ CR_WT_ groups. Scale, 100 μm; 50 μm for zoomed images. Each *dot* represents mean (*B* and *C*) or individual mouse (*E–G, I,* and *J*). Data shown are pooled values from a minimum of 3 biological repeats with 3 to 5 mice per group. Refer to Supplementary Table 1 for the exact number of mice used for experiments. *P* values were determined on data plotted as mean ± standard error of the mean using 2-tailed unpaired Student *t* test (*F, G*, and *I*), Mann–Whitney test (*J*), 2-way analysis of variance with Sidak’s post-test for multiple comparisons (*C* and *E*) and log-rank (Mantel–Cox) test (*D*). ns, not significant; ∗*P* < .05; ∗∗*P* < .01; ∗∗∗*P* < .001; ∗∗∗∗*P* < .0001.

Given that a history of infectious colitis conferred protection against subsequent chemically induced inflammation, we next asked whether a history of sterile colonic inflammation would similarly protect mice from CR infection. To this end, C57BL/6 mice were administered 2% DSS in drinking water for 7 days, followed by recovery on clean water, with untreated (UT) mice serving as controls (Figure 3*A*). Three weeks following recovery and return to baseline weight (∼40 days post DSS); mice were infected with 10^9^ CFUs of CR_WT_ (Figure 3*A* and *B*). As expected for resistant C57BL/6 mice,^2^ CR_WT_ infection in UT mice caused mild colitis without measurable weight loss (Figure 3*C*). In contrast, CR_WT_ infection in mice with a history of DSS treatment (DSS-Ψ) led to rapid weight loss beginning at 4 dpi, reaching approximately 14% by 10 dpi, and a significantly lower probability of survival (Figure 3*C* and *D*). Despite comparable bacterial shedding between UT and DSS-Ψ mice, the latter showed increased fecal water content and developed severe colitis, as evidenced by marked colonic shortening, and a higher colon weight-to-length ratio (Figure 3*E–I*). Histologic analysis of the distal colon revealed extensive epithelial damage and inflammation in infected DSS-Ψ mice, characterized by crypt loss, submucosal thickening, and greater immune cell infiltration, whereas infected UT mice exhibited mild pathology (Figure 3*J* and *K*). Immunofluorescence staining for E-cadherin revealed reduced E-cadherin signal in infected DSS-Ψ mice, consistent with barrier impairment (Figure 3*L*). These findings demonstrate that a primary episode of chemically induced colitis impairs epithelial resilience, rendering the host susceptible to subsequent CR infection.

### *Citrobacter rodentium*–Induced Disruption of Tight Junction Is Needed for Protection From Dextran Sodium Sulfate–Induced Colitis

**Figure 4:**
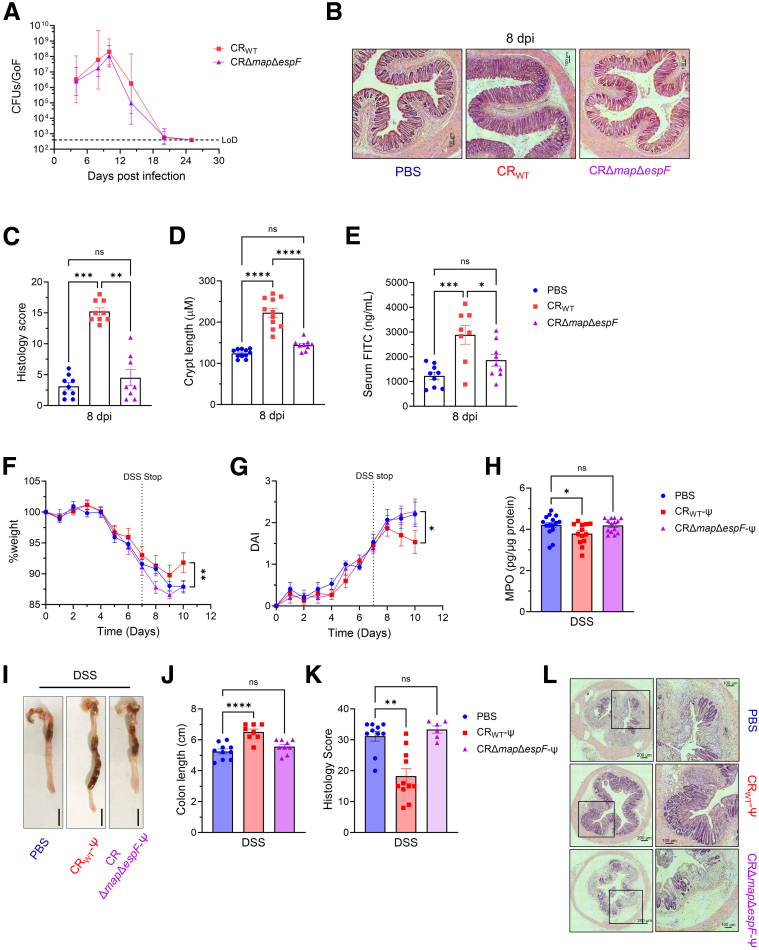
**Protection from colitis depends on CR infection-induced epithelial damage.** (*A–F*) Characterization of CRΔ*map*Δ*espF* infection at peak of infection (8 dpi). (*A*) Temporal fecal bacterial shedding following CR_WT_ and CRΔ*map*Δ*espF* infection. (*B*) Representative hematoxylin and eosin (H&E)-stained colon sections, (*C*) histologic scores for H&E-stained colon sections, and (*D*) measurement of crypt depth at 8 dpi following CR_WT_ and CRΔ*map*Δ*espF* infection. Scale, 200 μm. (*E*) Serum FITC concentration in infected mice at 8 dpi. (*F–L*) Assessment of DSS-induced colitis following prior CRΔ*map*Δ*espF* infection. (*F*) Weight change and (*G*) DAI in CR_WT_-Ψ, CRΔ*map*Δ*espF*-Ψ, or PBS mice, post DSS treatment. (*H*) Colonic MPO, (*I*) representative colon images, and (*J*) colon length. (*K*) Histologic scores and (*L*) representative H&E-stained colon sections. Scale, 200 μm. Each *dot* represents mean (*A, F*, and *G*) or individual mouse (*C–E, H, J*, and *K*). *Filled bars* represent data postsecondary challenge. Data shown are pooled values from a minimum of 3 biological repeats with 3 to 5 mice per group. Refer to Supplementary Table 1 for the exact number of mice used for experiments. Data shown are geometric mean ± standard deviation for (*A*). *P* values were determined on data plotted as mean ± standard error of the mean using 2-way analysis of variance (*F* and *G*), 1-way analysis of variance (*C–E, H*, and *J*) with Bonferroni post-test for multiple comparisons and Kruskal–Wallis test with Dunn’s correction (*K*). ns, not significant; ∗*P* < .05; ∗∗*P* < .01; ∗∗∗*P* < .001; ∗∗∗∗*P* < .0001.

Given the contrasting outcomes of secondary inflammation depending on whether the primary insult was infectious or sterile, we next sought to define the underlying mechanisms, starting with how CR_WT_-Ψ mice are protected from DSS-induced colitis. We first hypothesized that CR-mediated disruption of the barrier function of colon, which is driven by the type III secretion system effectors EspF and Map,^7^^,^^24^ would be essential for training the epithelium and for protection from the secondary DSS perturbation. To test this, we infected mice with CR lacking Map and EspF (CRΔ*map*Δ*espF*). Mice infected with CRΔ*map*Δ*espF* showed similar fecal bacterial shedding kinetics to CR_WT_ mice (Figure 4*A*). To confirm that CRΔ*map*Δ*espF* induces minimal colonic damage, mice were harvested at 8 dpi, corresponding to the peak of infection. Consistent with previous reports,^7^ CRΔ*map*Δ*espF*-infected mice exhibited reduced histopathologic damage and lacked CCH, a hallmark of CR infection (Figure 4*B–D*). To directly assess intestinal permeability, fluorescein isothiocyanate (FITC)-dextran assays were performed. Although CR_WT_-infected mice showed increased intestinal permeability, CRΔ*map*Δ*espF*-infected mice displayed no significant increase in serum FITC levels compared with uninfected controls (Figure 4*E*). Together, these findings indicate that CRΔ*map*Δ*espF* maintains comparable colonization while inducing minimal epithelial barrier disruption, making it a suitable tool to assess the contribution of epithelial injury to protection against DSS-induced colitis.

Mice with a history of CRΔ*map*Δ*espF* infection (CRΔ*map*Δ*espF*-Ψ) were equally susceptible to DSS-induced colitis as the control PBS mice (Figure 4*F* and *G*). CRΔ*map*Δ*espF*-Ψ mice had similar weight changes and DAI to PBS mice during DSS treatment, which was significantly different than CR_WT_-Ψ mice (Figure 4*F* and *G*). CRΔ*map*Δ*espF*-Ψ mice displayed similar colonic MPO levels and colonic shortening to PBS mice post DSS treatment, suggesting a similar extent of colonic damage and inflammation (Figure 4*H–J*). Histologic studies of distal colon revealed similar grade of colonic crypt architecture loss, immune cell infiltration, submucosal thickening, and epithelial disruption in PBS and CRΔ*map*Δ*espF*-Ψ mice (Figure 4*K* and *L*). Collectively, these results show that epithelial tight-junction disruption during primary CR infection is required for induction of protective responses against subsequent chemically induced colitis.

### *Citrobacter rodentium*–Ψ Mice Display an Increased Number of T Cells and Elevated Cytokine Levels in the Colon

**Figure 5:**
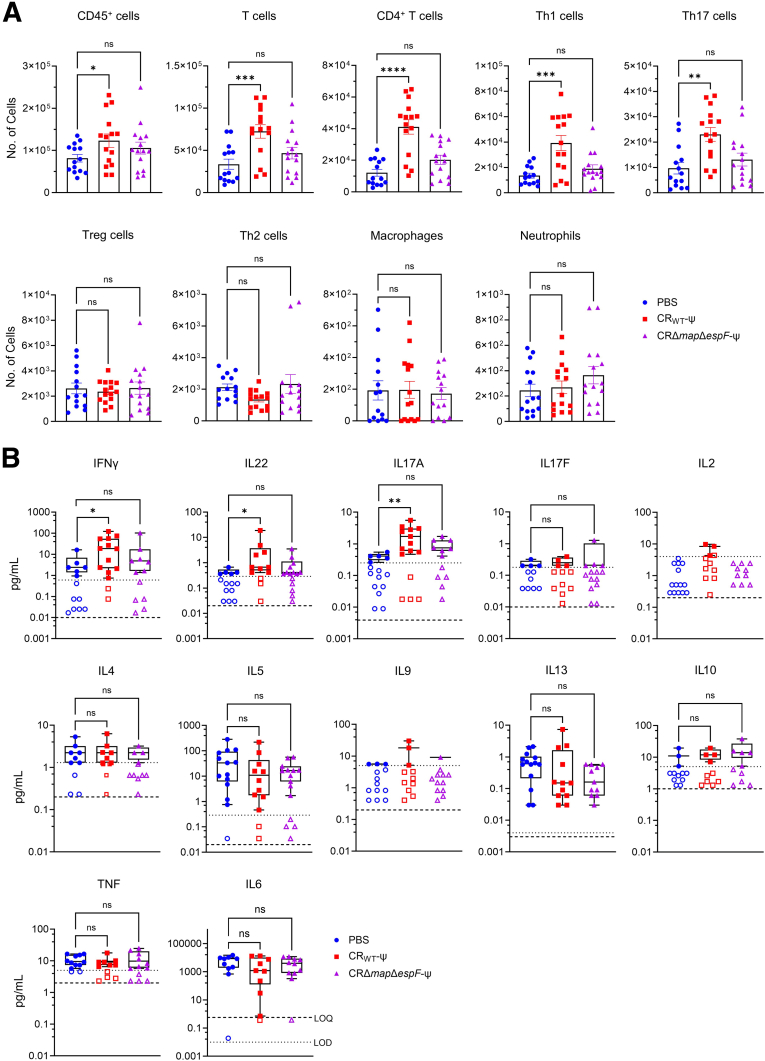
**Mice with a history of CR infection display an expanded T cell population.** (*A*) Flow cytometric quantification of CD45^+^ cells, CD45^+^ CD3^+^ total T cells, CD4^+^ T cells, Th1 cells, Th17 cells, Treg cells, Th2 cells, macrophages, and neutrophils in the colonic lamina propria at 40 dpi. (*B*) Cytokine analysis of colonic explant culture supernatants at 40 dpi for IFNγ, IL22, IL17A, IL17F, IL2, IL4, IL5, IL9, IL13, IL10, TNF, and IL6. Each *dot* represents an individual mouse. Data shown are pooled values from a minimum of 3 biological repeats with 3 to 5 mice per group. Refer to Supplementary Table 1 for the exact number of mice used for experiments. Dashed lines indicate analyte-specific limit of detection (LOD) and LOQ. All values above the LOD are shown; statistical analyses were performed only on values above the LOQ. *P* values were determined on data plotted as mean ± standard error of the mean using 1-way analysis of variance with Bonferroni post-test for multiple comparisons (*A*) and Kruskal–Wallis test with Dunn’s correction (*B*). ns, not significant; ∗*P* < .05; ∗∗*P* < .01; ∗∗∗*P* < .001; ∗∗∗∗*P* < .0001.

We next examined whether the protective imprint of CR infection against subsequent DSS-induced colitis was associated with long-term changes in the colonic immune landscape. The lamina propria of the colon was analyzed by flow cytometry at 40 dpi. CR_WT_-Ψ mice exhibited significantly higher numbers of CD45^+^ cells, total CD45^+^CD3^+^ T cells, and CD4^+^ T cells compared with PBS or CR*ΔmapΔespF*-Ψ mice, indicating persistent alterations in T cell abundance following infection (Figure 5*A*); neutrophils and macrophages counts were comparable among all groups (Figure 5*A*). Further characterization revealed no significant differences in regulatory T (Treg) or T helper 2 (Th2) cell populations, whereas CR_WT_-Ψ mice displayed an expansion of Th1 and Th17 cells relative to PBS and CR*ΔmapΔespF*-Ψ controls (Figure 5*A*). These results demonstrate that an expanded T cell compartment, dominated by T helper 1 (Th1) and Th17 cell subsets, persists in the colonic lamina propria after clearance of CR.

To determine whether these expanded populations remained functionally active, we next assessed cytokine production in colonic explant cultures at 40 dpi. CR_WT_-Ψ mice produced significantly higher levels of Th1-associated IFNγ compared with PBS or CR*ΔmapΔespF*-Ψ mice, whereas the level of IL2 was similar across groups (Figure 5*B*). In contrast, levels of type 2 cytokines IL4, IL5, IL9, and IL13 were similar across all groups, consistent with the unchanged Th2 cell frequencies (Figure 5*B*). Likewise, no differences were observed in Treg-derived IL10 or in the proinflammatory cytokines tumor necrosis factor (TNF) and IL6 (Figure 5*B*). Notably, CR_WT_-Ψ mice displayed markedly elevated levels of Th17-associated cytokines IL22 and IL17A compared with PBS or CR*ΔmapΔespF*-Ψ mice, whereas IL17F levels remained unchanged (Figure 5*B*). Together, these findings show that at 40 dpi, approximately 3 weeks after CR clearance, the colon harbors increased numbers of Th1 and Th17 cells, which are associated with sustained cytokine production.

### Interleukin17A Plays a Key Role in Mediating Protection From Dextran Sodium Sulfate–Induced Colitis in CR_WT_-Ψ Mice

Previous studies have established clear but opposing roles for IFNγ and IL22 during DSS-induced colitis. Mice lacking IFNγ are resistant to DSS-induced colitis, whereas exogenous IFNγ exacerbates disease severity,^25^^,^^26^ while IL22 protects against DSS-mediated injury by promoting epithelial regeneration.^19^ In contrast, the role of IL17A in intestinal inflammation remains context-dependent and less clearly defined, with studies reporting both pathogenic and protective functions.^21^^,^^22^ In line with this, IL17A has been shown to both induce neutrophil-recruiting chemokines and epithelial barrier–stabilizing molecules.^27^ Given this ambiguity, we investigated whether persistent IL17A signaling following CR clearance contributes to epithelial readiness and the protective phenotype against DSS-induced colitis.

**Figure 6:**
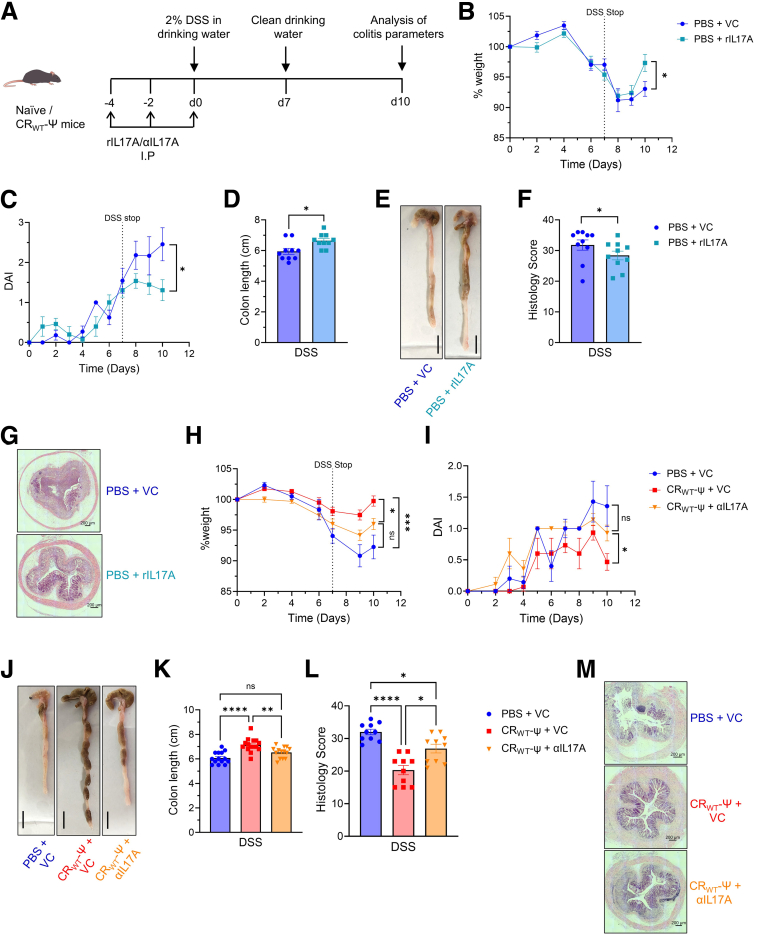
**IL17A contributes to protection from DSS-induced colitis.** (*A*) Schematic representation of the treatment. (*B–G*) Naïve mice were pretreated with rIL17A or vehicle control (VC) prior to DSS administration. (*B*) Weight change and (*C*) DAI post DSS treatment. (*D*) Colon length, (*E*) representative colon images, (*F*) histologic scores, and (*G*) representative hematoxylin and eosin (H&E)-stained colon sections. Scale, 200 μm. (*H–M*) CR_WT_-Ψ mice were treated with anti-IL17A neutralizing antibodies (αIL17A) or vehicle control (VC) prior to DSS administration. (*H*) Weight change and (*I*) DAI post DSS treatment. (*J*) Representative colon images, (*K*) colon length, (*L*) histologic scores, and (*M*) representative H&E-stained colon sections. Scale, 200 μm. Each *dot* represents mean (*B, C, H*, and *I*) or individual mouse (*D, F, K*, and *L*). Data shown are pooled values from a minimum of 3 biological repeats with 3 to 5 mice per group. Refer to Supplementary Table 1 for the exact number of mice used for experiments. *P* values were determined on data plotted as mean ± standard error of the mean using 2-tailed unpaired Student *t* test (*D* and *F*), 2-way analysis of variance (*B, C, H*, and *I*), 1-way analysis of variance with Bonferroni post-test for multiple comparisons (*K*), and Kruskal–Wallis test with Dunn’s correction (*L*). ns, not significant; ∗*P* < .05; ∗∗*P* < .01; ∗∗∗*P* < .001; ∗∗∗∗*P* < .0001.

To test this, naïve mice were administered recombinant IL17A (rIL17A), intraperitoneally prior to DSS treatment (Figure 6*A*); mice injected with vehicle were used as controls. Mice receiving rIL17A exhibited improved recovery following DSS exposure, as evidenced by greater post-treatment weight gain and a lower DAI (Figure 6*B* and *C*). Mice treatment with rIL17A displayed longer colons and reduced histologic inflammation and epithelial damage (Figure 6*D–G*). These findings indicate that exogenous IL17A can recapitulate key aspects of the protective phenotype observed in CR_WT_-Ψ mice.

To further assess the role of IL17A in CR infection–mediated protection from colitis, CR_WT_-Ψ mice were treated intraperitoneally with anti-IL17A antibodies prior to DSS treatment, with vehicle-treated CR_WT_-Ψ mice used as controls (Figure 6*A*). Anti–IL17A-treated CR_WT_-Ψ mice exhibited increased weight loss and higher DAI compared with vehicle-treated CR_WT_-Ψ mice following DSS treatment, consistent with a reduction in protection (Figure 6*H* and *I*). Anti–IL17A-treated CR_WT_-Ψ mice showed modest but nonsignificant differences in weight compared with PBS mice (Figure 6*H*). Similarly, no significant differences were observed in colon length between anti–IL17A-treated CR_WT_-Ψ and PBS mice, both of which were significantly shorter than CR_WT_-Ψ control mice (Figure 6*J* and *K*). Histologic analysis further revealed increased inflammation and epithelial damage in anti–IL17A-treated CR_WT_-Ψ mice compared with vehicle-treated CR_WT_-Ψ mice, although these remained lower than in PBS controls (Figure 6*L* and *M*). Together, these data indicate that IL17A contributes to the protection observed from DSS-induced colitis.

### Mice With a History of Dextran Sodium Sulfate–Induced Colitis Show Sustained T Cell Expansion

**Figure 7:**
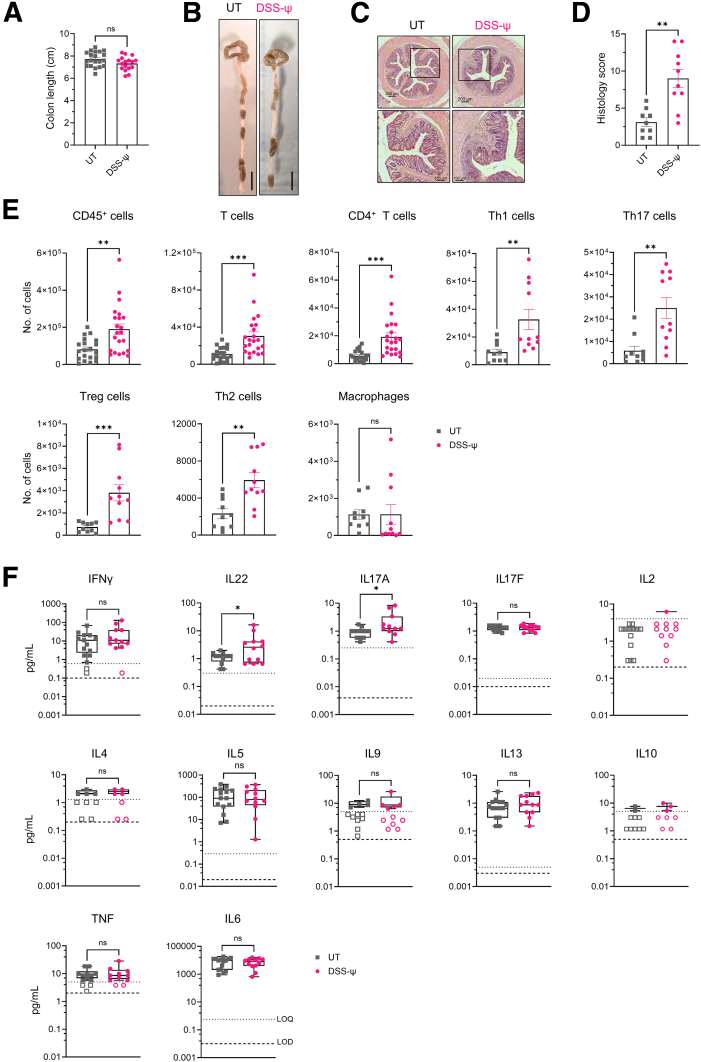
**Mice with a history of DSS display sustained expansion of colonic T cells.** C57BL/6 mice were treated with 2% DSS in drinking water, and 3 weeks post weight recovery (∼40 days post start of DSS treatment), mice (DSS-ψ) were harvested. UT mice were used as control. (*A*) Colon length, (*B*) representative colon images, (*C*) representative hematoxylin and eosin (H&E)-stained distal colon sections, and (*D*) histologic scores. Scale, 200 μm; 100 μm for zoomed images. (*E*) Flow cytometric quantification of CD45^+^ cells, CD45^+^ CD3^+^ total T cells, CD4^+^ T cells, Th1 cells, Th17 cells, Treg cells, Th2 cells, and macrophages in the colonic lamina propria. (*F*) Cytokine analysis of colonic explant culture supernatants for IFNγ, IL22, IL17A, IL17F, IL2, IL4, IL5, IL9, IL13, IL10, TNF, and IL6. Each *dot* represents an individual mouse. Data shown are pooled values from a minimum of 3 biological repeats with 3 to 5 mice per group. Refer to Supplementary Table 1 for the exact number of mice used for experiments. Dashed lines indicate analyte-specific limit of detection (LOD) and LOQ. All values above the LOD are shown; statistical analyses were performed only on values above the LOQ. *P* values were determined on data plotted as mean ± standard error of the mean using 2-tailed unpaired Student *t* test (*A* and *E*) and Mann–Whitney test (*D* and *F*). ns, not significant; ∗*P* < .05; ∗∗*P* < .01; ∗∗∗*P* < .001.

Given that protection in CR_WT_-Ψ mice was associated with sustained Th17 expansion and elevated IL17A, we next investigated these features in DSS-Ψ mice, which display susceptibility to CR infection. To this end, mice were culled 3 weeks after recovery (∼40 days after initiation of DSS treatment), and the distal colon was analyzed. Both UT and DSS-Ψ mice showed comparable colon lengths, consistent with apparent macroscopic recovery (Figure 7*A* and *B*). Histologic examination revealed restoration of crypt architecture in DSS-Ψ mice. However, DSS-Ψ mice displayed higher histologic scores, indicative of the mild residual inflammation, submucosal thickening, and epithelial disruption, suggesting incomplete resolution of inflammation (Figure 7*C* and *D*).

Flow cytometric analysis showed that, similarly to CR_WT_-Ψ mice, DSS-Ψ mice exhibited significantly greater numbers of CD45^+^ cells, total CD45^+^CD3^+^ T cells, and CD4^+^ T cells compared with UT controls (Figure 7*E*). Further characterization revealed increased frequencies of Th1, Th17, Treg, and Th2 subsets in DSS-Ψ mice relative to UT mice (Figure 7*E*). However, no differences were observed in the number of macrophages (Figure 7*E*).

Cytokine profiling of colonic explants showed that, despite increased T cell subsets, DSS-Ψ mice exhibited cytokine levels comparable to UT controls for Th1-associated IFNγ and IL2, Th2 cytokines (IL4, IL5, IL9, IL13), Treg-derived IL10, and proinflammatory mediators TNF and IL6 (Figure 7*F*). In contrast, similar to CR_WT_-Ψ mice, DSS-Ψ mice displayed significantly elevated levels of Th17-associated cytokines IL22 and IL17A relative to UT mice, with no change in IL17F (Figure 7*F*). Together, these results indicate that, similarly to CR_WT_-Ψ mice, DSS-Ψ mice display a persistently expanded T cell compartment and elevated IL22 and IL17A levels after clinical recovery from colitis.

### Persistent Neutrophil Accumulation and Epithelial Barrier Defects Distinguish DSS-Ψ Mice From CR_WT_-Ψ Mice

**Figure 8:**
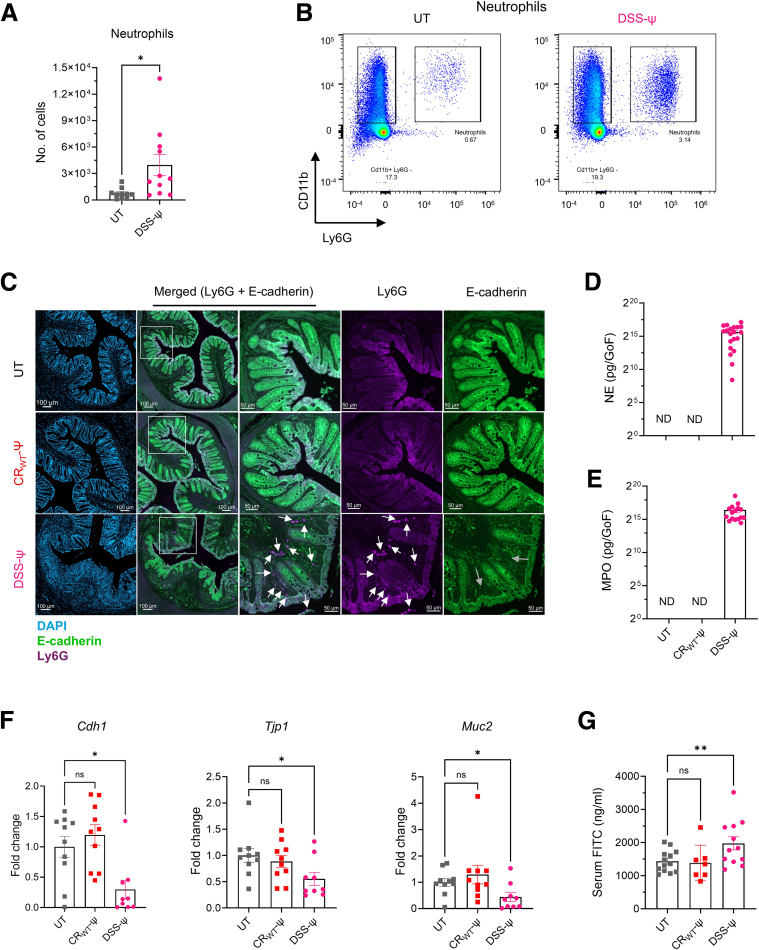
**Mice with a history of DSS display neutrophilia and epithelial barrier disruptio**n. (*A*) Flow cytometric quantification of CD45^+^ CD11b^+^ Ly6G^+^ neutrophils in the colonic lamina propria of DSS- ψ mice and (*B*) representative flow cytometry dot plots. (*C*) Representative immunostaining images of colonic sections for E-cadherin (*green*) and Ly6G^high^ (*violet*) from UT, CR_WT_-ψ, and DSS-ψ mice at 40 dpi or ∼40 days post start of DSS treatment, showing neutrophil influx to the colonic mucosa. 4',6-diamidino-2-phenylindole (DAPI; *blue*) was used for nuclei staining. *White arrows* indicate recruited neutrophils; *gray arrows* indicate erosion of colonic epithelial layer. Scale, 100 μm; 50 μm for zoomed images. (*D* and *E*) Fecal (*D*) NE and (*E*) MPO levels. (*F*) Quantitative reverse transcription polymerase chain reaction for fold change gene expression levels of *Cdh1*, *Tjp1*, and *Muc2* from distal colonic tissue compared with untreated controls. (*G*) Serum FITC-dextran levels for UT, CR_WT_-ψ, and DSS-ψ mice at 40 dpi or ∼40 days post start of DSS treatment, displaying persistent higher intestinal permeability in DSS-ψ mice. Each *dot* represents an individual mouse. Data shown are pooled values from a minimum of 3 biological repeats with 3 to 5 mice per group. Refer to Supplementary Table 1 for the exact number of mice used for experiments. *P* values were determined on data plotted as mean ± standard error of the mean using 2-tailed unpaired Student *t* test (*A*) and 1-way analysis of variance with Sidak’s post-test for multiple comparisons (*F* and *G*). ND, not detectable; ns, not significant; ∗*P* < .05; ∗∗*P* < .01.

Given the comparable T cell expansion and elevated IL22 and IL17A levels observed in DSS-Ψ and CR_WT_-Ψ mice, we next examined whether additional immune and epithelial features distinguish these groups. Flow cytometric analysis of the colon showed that, unlike CR_WT_-Ψ mice, DSS-Ψ mice had significantly higher numbers of neutrophils compared with UT controls (Figure 8*A* and *B*). Anti-Ly6G staining of distal colon sections corroborated these findings, showing markedly increased E-cadherin^−^ Ly6G^+^ neutrophils in DSS-Ψ mice, whereas UT and CR_WT_-Ψ mice displayed comparable staining (Figure 8*C*). Consistently, fecal neutrophil elastase (NE) and MPO levels were undetectable in CR_WT_-Ψ and UT mice at 40 dpi but were markedly elevated in DSS-Ψ mice (Figure 8*D* and *E*). These findings indicate that, although DSS-Ψ mice share an expanded Th17 compartment and elevated IL22 and IL17A levels similar to CR_WT_-Ψ mice, they uniquely harbor a persistent neutrophil infiltrate.

Assessment of intestinal barrier integrity further emphasized these differences. Immunofluorescence staining for E-cadherin showed intact epithelial integrity in UT and CR_WT_-Ψ mice but a markedly reduced E-cadherin signal in DSS-Ψ mice, consistent with impaired barrier function (Figure 8*C*). Analysis of transcript levels of epithelial integrity genes by quantitative reverse transcription polymerase chain reaction revealed that UT and CR_WT_-Ψ mice expressed comparable levels of *Tjp1*, *Muc2*, and *Cdh1*, whereas DSS-Ψ mice displayed significantly lower transcript abundance (Figure 8*F*). To directly evaluate the functional consequence of the impairment, FITC-dextran permeability assays were performed. Although CR_WT_-Ψ mice showed serum FITC levels comparable to UT controls, DSS-Ψ mice exhibited significantly higher serum FITC levels, indicating persistent barrier dysfunction (Figure 8*G*). Collectively, these data indicate that despite a shared Th17-rich cytokine environment, DSS-Ψ mice maintain chronic neutrophil infiltration and persistent epithelial barrier disruption that likely underlie their heightened susceptibility to CR infection.

### Unresolved Intestinal Epithelial Barrier Contributes to Susceptibility to *Citrobacter rodentium* Infection

Th17 cell expansion and the associated IL17A cytokine protected CR_WT_-Ψ mice from subsequent chemically induced colitis. However, despite similarly elevated Th17 populations and IL17A levels, DSS-Ψ mice remained highly susceptible to infection. Because higher neutrophil accumulation and persistent epithelial disruption were observed specifically in DSS-Ψ mice, we next examined whether the sustained neutrophilia contributed to their increased susceptibility to CR.

**Figure 9:**
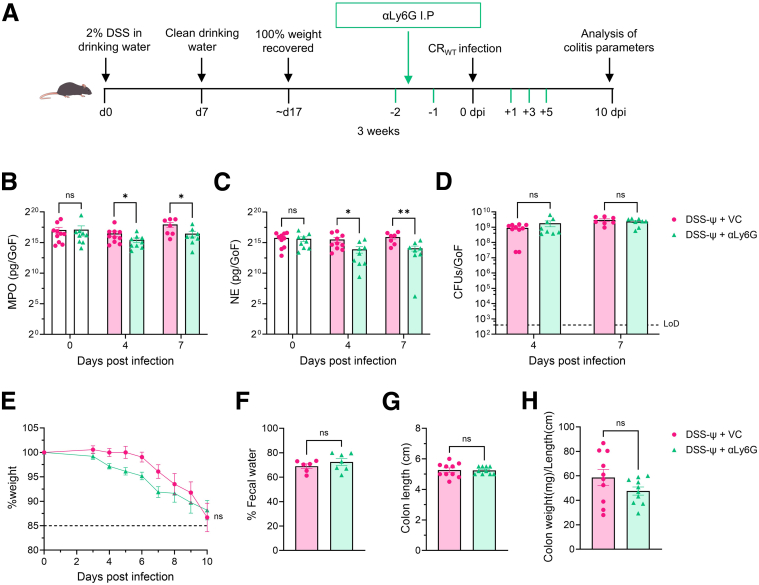
**Neutrophil depletion in DSS-Ψ mice does not rescue susceptibility to infectious colitis.** (*A*) Schematic representation of the treatment. DSS-ψ mice were treated with anti-Ly6G antibody (αLy6G) or vehicle control (VC) and infected with CR_WT._ (*B* and *C*) Fecal (*B*) MPO and (*C*) NE levels at 0, 4, and 7 dpi. (*D*) Temporal fecal bacterial shedding. (*E*) Weight loss post CR_WT_ infection. (*F*) Fecal water content at 7 dpi. (*G*) Colon length and (*H*) colon weight-to-length ratio of mice harvested at 10 dpi. Each *dot* represents mean (*E*) or individual mouse (*B–D, F–H*). *Filled bars* represent data postsecondary challenge. Data shown are pooled values from a minimum of 3 biological repeats with 3 to 5 mice per group. Refer to Supplementary Table 1 for the exact number of mice used for experiments. *P* values were determined on data plotted as mean ± standard error of the mean using 2-way analysis of variance with Sidak’s post-test for multiple comparisons (*B–E*) and 2-tailed unpaired Student *t* test (*F–H*). ns, not significant; ∗*P* < .05; ∗∗*P* < .01.

To test this, DSS-Ψ mice were treated intraperitoneally with anti-Ly6G antibodies from 2 days before infection until 5 dpi to deplete neutrophils; vehicle-treated DSS-Ψ mice were used as controls (Figure 9*A*). Anti–Ly6G-treated mice displayed markedly reduced fecal MPO and NE levels (Figure 9*B* and *C*), confirming effective neutrophil depletion. However, depletion did not alter subsequent CR infection outcome; anti-Ly6G-treated DSS-Ψ mice exhibited weight loss, bacterial shedding, and fecal water content comparable to vehicle-treated DSS-Ψ mice (Figure 9*D–F*). Colon length and colon weight-to-length ratios were also similar between groups, indicating that anti-Ly6G treatment did not ameliorate disease severity (Figure 9*G* and *H*).

**Figure 10:**
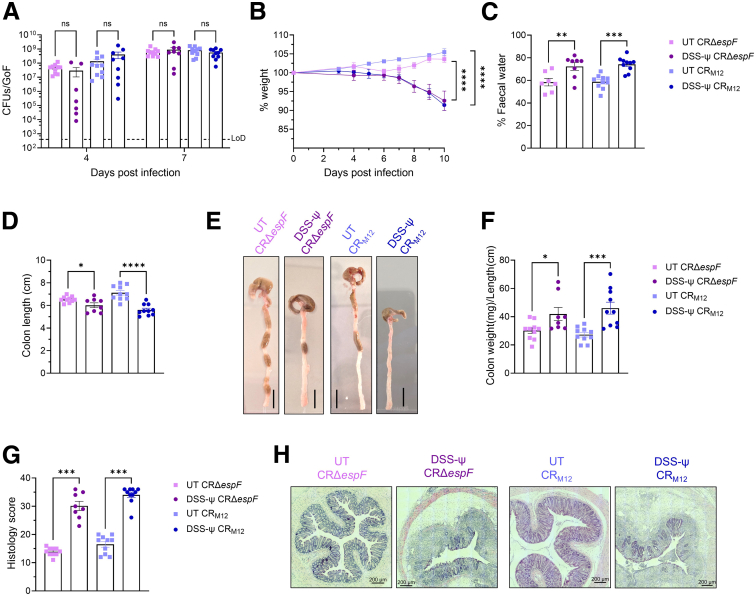
**Unresolved epithelial barrier dysfunction contributes to susceptibility to CR infection.** DSS-ψ mice were infected with either CRΔ*espF* or CR_M12_. UT mice were infected with respective mutants and used as controls. (*A*) Temporal fecal bacterial shedding following infection. (*B*) Weight loss, (*C*) fecal water content at 7 dpi, (*D*) colon length, (*E*) representative colon images, and (*F*) colon weight-to-length ratio postinfection. (*G*) Histologic scores and (*H*) representative hematoxylin and eosin (H&E)-stained colon sections from mice harvested at 10 dpi. Scale, 200 μm. Each *dot* represents mean (*B*) or individual mouse (*A, C, D, F*, and *G*). Data shown are pooled values from a minimum of 3 biological repeats with 3 to 5 mice per group. Refer to Supplementary Table 1 for the exact number of mice used for experiments. *P* values were determined on data plotted as mean ± standard error of the mean using 1-way analysis of variance (*A* and *B*) and 1-way analysis of variance (*C, D*, and *F*) with Sidak’s post-test for multiple comparisons, and Kruskal–Wallis test with Dunn’s correction (*G*). ns, not significant; ∗*P* < .05; ∗∗*P* < .01; ∗∗∗*P* < .001; ∗∗∗∗*P* < .0001.

We next tested whether unresolved epithelial barrier damage contributes to the heightened susceptibility of DSS-Ψ mice to CR infection. To this end, we used 2 CR mutant strains that cause mild disease in WT mice but result in distinct outcomes in the susceptible *Il22*^*-/-*^ mice. CRΔ*espF* causes limited epithelial barrier disruption and results in nonlethal infection in *Il22*^*-/-*^ mice.^28^ However, when the epithelial barrier is disrupted by CR_WT_ infection, CRΔ*espF* is systemically disseminated and results in fatal disease, making it a useful tool to assess existing barrier disruption.^28^ In contrast, CR_M12_, which lacks 12 effector genes, causes lethal infection in *Il22*^*-/-*^ mice, where epithelial repair is impaired, and therefore serves as a model to assess susceptibility driven by defective epithelial repair.^6^ Importantly, both mutant strains cause only mild disease in WT mice; therefore, they provide complementary tools to identify susceptibility driven by barrier disruption and impaired repair. We therefore hypothesized that if defective epithelial integrity and repair underlie susceptibility, DSS-Ψ mice would remain vulnerable to both mutant strains. Consistent with this hypothesis, DSS-Ψ and UT mice displayed similar colonization levels of CRΔ*espF* and CR_M12_ (Figure 10*A*). However, DSS-Ψ mice infected with either mutant developed severe disease, characterized by pronounced weight loss, increased fecal water content, significant colon shortening, and elevated colon weight-to-length ratios, reflecting inflammation (Figure 10*B–F*). Histologic analysis revealed marked epithelial erosion, crypt damage, and submucosal thickening in DSS-Ψ mice infected with CRΔ*espF* or CR_M12_, whereas UT mice infected with the same strains showed only mild crypt hyperplasia without epithelial erosion as reported earlier^6^^,^^28^ (Figure 10*G* and *H*). Together, these results demonstrate that unresolved epithelial barrier disruption contributes to the susceptibility to subsequent CR infection in mice with a history of chemical-induced colitis.

### DSS-Ψ Mice Exhibit Persistent Barrier Dysfunction and Susceptibility to *Citrobacter rodentium* Infection Following Extended Recovery

**Figure 11:**
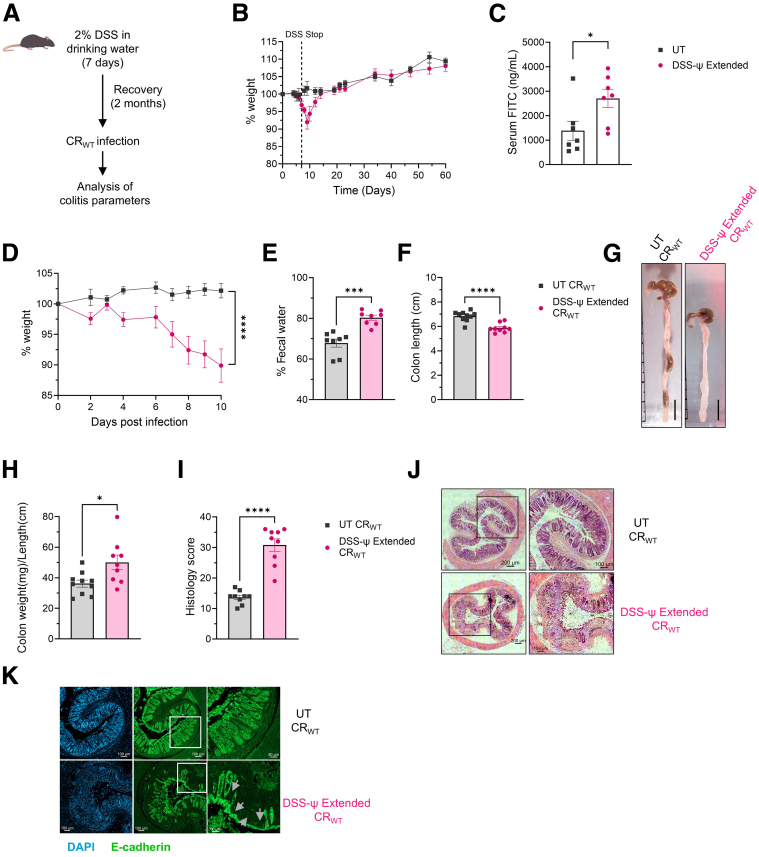
**Mice with a history of DSS display persistent vulnerability to CR infection following extended recovery.** (*A*) Schematic representation of the experimental design: C57BL/6 mice were treated with 2% DSS in drinking water, and 6 weeks post weight recovery (DSS-ψ Extended) were infected with CR_WT_. DSS-untreated mice, infected with CR_WT_ (UT CR_WT_) were used as control. (*B*) Percentage weight change post DSS treatment. (*C*) Serum FITC concentration assessing intestinal barrier integrity in mice with extended recovery. (*D*) Weight loss post CR_WT_ infection. (*E*) Fecal water content at 7 dpi. (*F*) Colon length, (*G*) representative colon images, (*H*) colon weight-to-length ratio, (*I*) histologic score, and (*J*) representative hematoxylin and eosin (H&E)-stained distal colon sections of mice at 10 dpi. (*K*) Representative immunostaining images of colonic sections from CR_WT_-infected UT and DSS-ψ extended mice at 10 dpi. Scale, 200 μm. The IECs express E-cadherin (*green*) and are 4',6-diamidino-2-phenylindole (DAPI^+^; *blue*). In the zoomed E-cadherin panel, *gray arrows* indicate erosion of colonic epithelial layer, and loss of colonic crypt architecture represented by lower/loss of E-cadherin staining in DSS-ψ CR_WT_ groups. Scale, 200 μm; 100 μm for zoomed images. Each dot represents mean (*B* and *D*) or individual mouse (*C, E, F, H*, and *I*). *Filled bars* represent data postsecondary challenge. Data shown are pooled values from a minimum of 3 biological repeats with 3 to 5 mice per group. Refer to Supplementary Table 1 for the exact number of mice used for experiments. *P* values were determined on data plotted as mean ± standard error of the mean using 2-way analysis of variance (*D*), 2-tailed unpaired Student *t* test (*C, E, F*, and *H*) and Mann–Whitney test (*I*). ns, not significant; ∗*P* < .05; ∗∗∗*P* < .001; ∗∗∗∗*P* < .0001.

We next asked whether the impaired intestinal epithelial barrier integrity and increased susceptibility to CR infection resolve with extended recovery from the initial DSS administration. To address this, DSS-treated mice were infected with CR_WT_ 6 weeks after regaining baseline weight (∼2 months post DSS) (Figure 11*A* and *B*). Assessment of intestinal permeability using FITC-dextran assays showed that DSS-Ψ mice continued to exhibit higher serum FITC levels compared with untreated controls, indicating persistent epithelial barrier dysfunction (Figure 11*C*). Consistent with this, upon secondary CR_WT_ challenge, DSS-Ψ mice with extended recovery developed pronounced disease. These mice exhibited significant weight loss, increased fecal water content, shortened colons, and elevated colon weight-to-length ratios, indicative of inflammation (Figure 11*D–H*).

Histologic and immunofluorescence analyses further showed that, similarly to mice assessed after 3 weeks of recovery, DSS-Ψ mice examined after 6 weeks continued to display extensive epithelial injury following CR_WT_ infection. Colonic sections revealed submucosal thickening, crypt loss, epithelial erosion, and markedly reduced E-cadherin staining (Figure 11I–K). Together, these findings indicate that a primary episode of chemically induced colitis is associated with persistent impairment of epithelial barrier integrity, which contributes to sustained susceptibility to subsequent CR infection.

Collectively, these results indicate that the nature, context, and epithelial consequences of the primary inflammatory episode shape whether prior intestinal inflammation is associated with a protective or pathologic mucosal state.

## Discussion

The interplay between prior intestinal inflammation and the immune system in determining susceptibility to future disease is complex and remains incompletely understood. In this study, we show that resolution of a CR infection confers durable protection against chemically induced colitis, whereas a history of DSS-induced colitis predisposes mice to heightened susceptibility to subsequent CR infection. These findings suggest that the nature and context of the primary inflammatory insult influence whether prior disease history exerts a protective or detrimental effect on future mucosal inflammation.

CR infection induces durable inflammatory memory within the colonic microenvironment, characterized by elevated IFNγ, increased epithelial MHCII expression, and expansion of enteroendocrine cells.^14^ It also generates long-lived ILC3s that exhibit enhanced proliferative capacity and augmented IL22 responses, providing superior control of infection compared with naïve ILC3s.^10^ Likewise, DSS treatment produces epithelial and mucosal abnormalities that persist for several weeks beyond apparent recovery.^17^ Three weeks after DSS withdrawal, colonic histology reveals residual ulceration and mucosal erosion, accompanied by elevated expression of *Il1b*, *Tnf*, and *Il33* transcripts and sustained serum amyloid A levels, indicative of persistent low-grade inflammation.^17^ Mice recovered from both CR infection and DSS-induced colitis also harbor microbiota-dependent, long-lived, tissue-resident CD44^+^CD69^+^ Th17 cells with a tissue-resident memory phenotype, which confer protection against subsequent DSS-mediated colitis.^13^

Mice with a history of CR infection were protected from subsequent DSS-induced colitis, whereas mice that had resolved *Salmonella typhimurium*^29^ or *Toxoplasma gondii*^30^ infections exhibited increased susceptibility to DSS-induced injury. Protection mediated by CR is dependent on induction of epithelial injury during the primary infection. Mice infected with the CR*ΔmapΔespF* mutant, which induces minimal tight-junction disruption and CCH, reduced immune cell infiltration, and lower proinflammatory cytokine production.^7^ failed to protect against subsequent colitis. Although neutrophil, dendritic cell, B cell, and CD4^+^ T cell recruitment are known to be comparable between CR*ΔmapΔespF* and CR at the peak of infection (8 dpi),^7^ sustained CD4^+^ T cell expansion following bacterial clearance appeared to depend on sufficient tight-junction disruption and epithelial barrier damage.

Mice with a prior history of either CR infection or DSS-induced colitis exhibited sustained elevations in colonic Th17 cells accompanied by increased expression of IL22 and IL17A. These lymphocytes persisted in the lamina propria beyond disease resolution, indicating sustained alterations in mucosal immune populations. Persistent IL17A in mice with a history of CR infection contributed to protection against chemically induced colitis. Administration of rIL17A markedly attenuated disease severity following DSS treatment, whereas neutralization of IL17A in CR-experienced mice reduced this protection. In contrast, mice recovered from DSS-induced colitis did not exhibit similar protection upon subsequent CR infection despite displaying elevated IL17A levels. Persistent Th17 cells and their IL17A secretion after DSS treatment have been implicated in heightened susceptibility to *C difficile* infection.^18^ Similarly, *Blastocystis* ST7 colonization enriches IL17A/TNF-producing CD4^+^ T cells that exacerbate DSS-induced colitis,^31^ underscoring the context-dependent and sometimes deleterious effects of elevated IL17A.

The role of IL17A in colonic inflammation has long been debated.^27^ Early studies suggested a pathogenic function, as *Il17a*-deficient mice were protected from DSS-induced colitis.^32^ In contrast, later work using *Il17a*-deficient mice or IL17A blockade demonstrated a protective role, showing that IL17A supports epithelial repair by strengthening tight-junction integrity, promoting epithelial proliferation, and facilitating mucosal wound healing.^22^ Human clinical trials mirror this complexity, where anti-IL17A therapies that are beneficial in autoimmune diseases such as psoriasis have paradoxically worsened outcomes in patients with IBD,^21^^,^^33^, ^34^, ^35^ highlighting the tissue-specific nature of IL17A function.

Notably, Th17 cells elicited by CR infection differ fundamentally from those expanded by commensals such as segmented filamentous bacteria.^36^ Infection-induced Th17 cells exhibit pronounced cytokine plasticity, disseminate systemically, and display features of inflammatory effector cells. By contrast, commensal-induced Th17 cells are metabolically quiescent, show limited cytokine flexibility, and rarely contribute to inflammation.^36^ These distinctions may help explain why IL17A induced during CR infection promotes mucosal protection, whereas IL17A persisting after DSS-induced colitis fails to confer similar benefit.

Mice recovered from DSS-induced colitis exhibited persistent epithelial barrier disruption and colonic neutrophilia. Neutrophils are rapidly recruited to the colon during CR infection and play important roles in bacterial containment and mucosal defense.^37^ Consistent with this, neutrophil depletion using anti-Ly6G antibodies or impaired neutrophil recruitment in CXCR2-deficient mice results in uncontrolled CR infection, severe intestinal inflammation, and increased mortality.^37^^,^^38^ However, excessive neutrophil accumulation and activation has also been linked to epithelial injury, colitis, and mortality in susceptible hosts.^39^ Increased colonic NE activity following CR infection in C3H/HeN mice is associated with heightened epithelial and tissue damage, highlighting the potential of neutrophils to drive pathologic inflammation.^39^ Similarly, increased neutrophil infiltration after DSS administration has been linked to greater susceptibility to *C difficile* infection.^40^

In our study, although DSS-Ψ mice exhibited persistent neutrophilia, their heightened vulnerability to CR infection was not solely attributable to neutrophil accumulation but was associated with incomplete epithelial barrier repair. This interpretation is supported by the susceptibility of mice with a history of DSS-induced colitis to both CRΔ*espF* and CR_M12_ mutant strains. CRΔ*espF* does not disrupt the epithelial barrier during infection.^28^ Consistently, IL22-deficient mice survive infection with CR*ΔespF*.^28^^,^^41^ Conversely, epithelial injury induced by CR infection renders IL22-deficient mice susceptible to CR*ΔespF*,^28^ highlighting the importance of barrier integrity in shaping infection outcomes.

Despite inducing less epithelial damage in WT mice, the CR_M12_ strain nevertheless causes severe disease in the susceptible IL22-deficient mice.^6^ CR_M12_ lacks 12 effectors that are dispensable for colonic colonization, forming an effector network that limits epithelial injury yet remains pathogenic when epithelial barrier repair is compromised.^6^ Mice recovered from DSS-induced colitis remain susceptible to CR_M12_ infection, further supporting a role for impaired epithelial repair in vulnerability to infection. Supporting this concept, prior colonization with adherent-invasive *E coli*, which impairs epithelial integrity and mucosal restitution, similarly increases susceptibility to CR infection and results in persistent CCH and sustained loss of goblet cells following bacterial clearance.^42^ Defects in epithelial barrier integrity may further exacerbate susceptibility by increasing mucosal exposure to luminal antigens, thereby sustaining immune activation.^43^ Consistent with this, reduced mucin production due to goblet cell depletion and epithelial tight junction disruption can facilitate antigen access to the intestinal mucosa and have been implicated in the pathophysiology of IBD.^44^

Mice recovered from DSS-induced colitis exhibited persistently elevated intestinal permeability and increased susceptibility to CR infection up to 60 days after treatment. This persistent defect underscores the long-term consequences of severe epithelial injury and may parallel observations in patients with IBD, who frequently experience disease flares or exacerbated symptoms following gastrointestinal infections despite apparent clinical remission.^45^^,^^46^ These findings highlight the enduring impact of prior colitis on epithelial resilience and host defense, emphasizing the need to better understand mechanisms that restore and maintain barrier integrity after inflammation.

In addition to host epithelial and immune factors, alterations in the gut microbiota may also influence susceptibility to secondary insults.^47^ Antibiotic exposure of young mice leads to persistent alterations in microbial communities, including reduced abundances of anaerobes (eg, *Bacteroidetes* and *Tenericutes*) and expansion of *Proteobacteria*, which are associated with increased susceptibility to CR infection even several weeks later.^47^ Similarly, the severity of DSS-induced colitis is influenced by microbiota composition, with differences observed between mice from different vendors, and short-chain fatty acid–producing communities generally associated with reduced disease severity.^48^

Both CR infection and DSS-induced colitis are known to induce gut microbiota dysbiosis.^49^, ^50^, ^51^ During peak CR infection, changes in IEC bioenergetics promote oxygenation of the mucosal surface, supporting the expansion of mucosal-associated *γ-proteobacteria* and a reduction in obligate anaerobes such as *Clostridia*.^52^ CR infection has also been associated with expansion of *Enterobacteriaceae*,^49^ although spatial analyses of luminal and mucosa-associated microbiota have reported more nuanced changes across intestinal regions and timepoints, including increased abundance of *Deferribacteres* and reduced abundance of *Lactobacillus*.^50^ DSS-induced colitis similarly results in marked microbial shifts, including reductions in *Bacteroidetes*/*Prevotella* and increases in *Bacillaceae* during active inflammation, with a general trend towards recovery following DSS withdrawal.^51^ These observations suggest that, although microbiota alterations likely contribute to the postcolitis mucosal environment, our data identify epithelial barrier integrity and IL17A-associated responses as major factors shaping outcomes in this model.

Together, these findings suggest that the nature, context, and pathologic consequences of the primary inflammatory insult influence whether prior disease history is associated with adaptive or maladaptive mucosal states. Our data support 2 complementary models that illustrate how prior intestinal inflammation can lead to divergent outcomes in mucosal homeostasis and disease susceptibility. These findings highlight epithelial barrier integrity and immune responses as key components of this process, providing a framework for understanding how prior inflammation shapes subsequent immune–epithelial interactions. Future studies aimed at restoring durable epithelial integrity and defining how infection-primed T cells and innate lymphoid populations are maintained will inform therapeutic strategies to enhance barrier resilience and reduce infection-associated flares in IBD.

## Materials and Methods

All the materials and key resources can be found in the file “Reagent and Resources table” on Figshare: https://doi.org/10.6084/m9.figshare.30601727. All authors had access to the study data and have reviewed and approved the final manuscript.

### Ethical Statement

All animal experiments were performed at Imperial College London in facilities accredited by the Association for Assessment and Accreditation of Laboratory Animal Care. Procedures were carried out in accordance with the UK Animals (Scientific Procedures) Act 1986 (Project License PP7392693 and PP8896560) and approved by the institutional Animal Welfare and Ethical Review Body.

### Mouse Experiments

Mouse experiments were performed in accordance with the Animals Scientific Procedures Act of 1986 and were approved by the local Ethical Review Committee according to UK Home Office guidelines. Specific pathogen-free, 18 to 20 g, female, C57BL/6 mice were purchased from Charles River Laboratories and housed in groups of 5 in dedicated animal facilities of Imperial College London (12-hour light/dark cycle; 22 °C ± 2 °C; 30%–40% humidity). Mice were housed in individually ventilated cages with corn cob bedding and enrichments including refuges, nesting material, and gnawing sticks. Mice were fed with RM1(E) rodent diet (SDS Diet) and water ad libitum.

### *Citrobacter rodentium* Infection and Dextran Sodium Sulfate–Induced Colitis

CR was grown overnight in lysogeny broth containing 50 μg/mL nalidixic acid at 37 °C with shaking at 180 rpm, centrifuged at 3000 g for 10 minutes and resuspended in sterile PBS.^53^ Mice were infected with approximately 3 × 10^9^ CFU in 200 μL sterile PBS using oral gavage, as previously described.^53^ Mock infected (PBS) mice received 200 μL sterile PBS. The inoculum CFU was retrospectively confirmed by CFU quantification. Mice were monitored every day. Body weight and CFU counts were assessed as previously described.^53^ Briefly, analysis of CFUs was determined via serial dilutions of homogenized fecal pellets on the indicated days post CR infection, followed by plating on lysogeny broth agar plates supplemented with 50 μg/mL nalidixic acid.

To assess the effect of CR history on DSS-induced colitis, at 40 dpi, mice were weighed and then administered with 2% DSS in drinking water for 7 days, followed by normal drinking water for 3 days. Mice were monitored every day for changes in weight and disease severity. To evaluate the severity of colitis, the DAI score was monitored daily. The DAI score was determined based on the methods described by Friedman et al.^54^ Briefly, the DAI score was calculated as the sum of the weight loss score, the diarrheal score, and the hematochezia score.

To determine the effect of rIL17A treatment on outcomes of DSS-induced colitis, naïve mice were treated with 2 μg of rIL17A intraperitoneally on −4, −2, and 0 days before the start of DSS administration. For anti-IL17A treatment, CR_WT_ infected mice post clearance (CR_WT_-Ψ) were injected with 100 μg of anti-IL17A antibody intraperitoneally on −4, −2, and 0 days before the start of DSS administration.

To assess the effect of DSS-induced colitis history on subsequent CR infection, mice were weighed and then administered with 2% DSS in drinking water for 7 days, followed by normal drinking water for 3 days (DSS-ψ mice). Mice were weighed every day for changes in weight. Three weeks after all mice recovered lost weight due to DSS treatment (∼40 days post start of DSS treatment), DSS-ψ and UT mice were infected with CR.

To deplete the increased neutrophil recruitment in DSS-ψ mice, mice were injected with 100 μg of anti-Ly6G antibody intraperitoneally on −2, −1, 1, 3, and 5 days post CR_WT_ infection.

### Postmortem Pathophysiological Analysis

Mice were humanely euthanized. The large intestine of mouse consisting of cecum and colon was harvested and placed on a clean surface in parallel to a scale, and the colon length was recorded and captured using a digital camera. The colon was removed from the cecum, cleaned, and from the distal side of colon, 0.5 cm of colon was stored in 4% paraformaldehyde for histologic studies; the next 0.5 cm was collected for RNA extraction, the next 0.5 cm was collected for explant culture, and the next 0.5 cm was snap-frozen in dry ice for MPO analysis.

### Cytokine Profiling From Explants

A 0.5-cm segment of distal colon, cleared of fecal matter, was weighed and incubated in 1 mL RPMI 1640 medium supplemented with glutamine (Sigma), streptomycin (100 μg/mL), and penicillin (100 U/mL) for 2 hours at room temperature. After this initial incubation, tissue explants were transferred to complete RPMI medium (containing glutamine, 10% heat-inactivated fetal bovine serum [FBS], 1 mM sodium pyruvate, 100 μg/mL penicillin, 100 μg/mL streptomycin, and 10 mM HEPES) at a concentration of 100 μL per 10 mg of tissue. Explants were cultured under standard conditions (37 °C, 5% CO_2_) for 24 hours. After incubation, the supernatants were collected, centrifuged at 3000 g for 10 minutes to remove residual cells and debris, and stored at −80 °C until analysis.

Cytokine concentrations in the explant supernatants were measured using the highly sensitive LEGENDplex MU Th Cytokine Panel kit following the manufacturer’s protocol (Catalogue No. 741044) as described earlier.^9^ Data acquisition was carried out on a FACSCalibur flow cytometer (BD Biosciences), and cytokine levels were quantified using the LEGENDplex data analysis software (BioLegend). Standard curves were generated for each analyte using a 5-parameter logistic regression model, and assay-specific limits of detection and lower limits of quantification (LOQs) were determined from the standard curve for each experimental run. All measured values above the limit of detection are presented for transparency. However, only values above the analyte-specific LOQ were considered reliably quantifiable and were included in statistical analyses. Values falling below the LOQ were excluded from statistical comparisons and interpreted with caution. This approach was applied consistently across all cytokines analyzed in the study.

### Histologic Analysis

Paraformaldehyde-fixed 0.5-cm distal colon samples were processed, paraffin-embedded, and sectioned at 5 μm.^53^ The sections were then stained with H&E or processed for immunofluorescence. Histopathologic scoring was performed as described earlier.^9^ Briefly, 4 parameters were evaluated in each section: (1) extent of epithelial damage; (2) immune cell infiltration; (3) loss of crypt architecture; and (4) percentage of area affected. Each was scored on a scale of 0 to 10, with 0 representing normal histology.

For immunofluorescence, sections were dewaxed by incubating in Histoclear solution for 10 minutes 2 times, followed by immersion in 100% ethanol for 10 minutes 2 times, 95% ethanol for 3 minutes 2 times, 80% ethanol for 3 minutes, and PBS-TS (1× PBS-0.1% Tween-20-0.1% saponin), for 3 minutes 2 times. The sections were heated for 30 minutes in demasking solution (0.3% trisodium citrate-0.05% Tween-20 in distilled H_2_O). After cooling, the slides were washed in PBS-TS, followed by blocking in PBS-TS supplemented with 10% normal donkey serum for 20 minutes. The slides were incubated overnight at 4 °C with anti-CR rabbit polyclonal antibody (1:50), mouse anti–proliferating cell nuclear antigen antibody (1:500), anti–E-cadherin (1:50), and/or anti-Ly6G (1:300) antibody. The following day, the slides were washed in PBS-TS, 10 minutes 2 times, and incubated with the appropriate secondary antibody (1:100) or 4',6-diamidino-2-phenylindole (1:1000) to stain DNA. The slides were washed and mounted with ProLong Gold antifade mountant. All images were acquired using a Zeiss AxioVision Z1 microscope with a 20× or 40× lens objective using an AxioCam MRm or Hamamatsu camera and processed using Zen 2.3 (Blue version; Carl Zeiss MicroImaging GmbH).

### Fecal Water Content Analysis

Fecal water content was estimated by the following method: feces were freshly collected in preweighed 1.5-mL tubes with punctured caps. The tubes containing wet feces were weighed and then incubated at 55 °C. The tubes were weighed daily until the weight did not change, and the weights were recorded. The wet weight and dry weight of feces was determined by subtracting the weight of the empty tube, and the fecal water content was estimated using the following equation:

$$\%\;water\;content=\frac{wet\;weight-dry\;weight}{wet\;weight}\;x\;100$$

### Colonic and Fecal Sample Enzyme-Linked Immunosorbent Assay

Frozen colon samples (0.5 cm) were mechanically homogenized in 500 μL of 1× radioimmunoprecipitation assay buffer containing protease inhibitors, followed by centrifugation at 3000 g for 10 minutes at 4 °C. The supernatant was collected, and the total protein concentration was estimated using a bicinchoninic acid kit. The MPO concentration was determined using a mouse MPO enzyme-linked immunosorbent assay (ELISA) according to the manufacturer’s instructions. Readings were obtained using a FLUOstar Omega microplate reader (BMG Biotech). The amount of MPO estimated was normalized to the protein concentration.

For ELISA using fecal samples, wet feces were collected, weighed, suspended in PBS + 0.1% Triton X-100 (1 mL per 100 mg wet feces) and homogenized for ∼10 minutes. Following a brief centrifugation, the supernatant was stored at −80 °C. The concentrations of fecal MPO and NE were determined using DuoSet mouse MPO and NE ELISA (R&D Systems) according to the manufacturer’s recommendations.

### Isolation of Lamina Propria Immune Cells From Mouse Colon

Lamina propria cells were isolated from mouse colons using a previously established protocol.^11^ Briefly, 4-cm segments of distal colon were excised, washed thoroughly, and opened longitudinally. The tissues were then incubated at 37 °C for 20 minutes in a shaking incubator in calcium- and magnesium-free 1× Hanks’ balanced salt solution containing 2% FBS, 10 mM EDTA, and 1 mM dithiothreitol to remove the epithelial layer. Following incubation, the cell suspension was centrifuged to separate IECs from the remaining lamina propria tissue. Supernatants containing IECs were discarded, and the residual tissue was subjected to enzymatic digestion in RPMI 1640 medium containing 62.5 μg/mL Liberase, 50 μg/mL DNase I (Sigma-Aldrich), and 2% FBS at 37 °C for 40 to 50 minutes. The resulting cell suspension was passed through a 100-μm cell strainer to obtain a single-cell suspension for downstream flow cytometry analysis.

### Flow Cytometry

For extracellular staining, single-cell suspensions were distributed into 96-well V-bottom plates and incubated for 10 minutes with LIVE/DEAD Fixable Blue dye (diluted in D-PBS) to identify and exclude nonviable cells from analysis. Following this, cells were incubated for 20 minutes with Fcγ receptor blocking reagent (BD Biosciences) to prevent nonspecific antibody binding and then stained for surface antigens using fluorochrome-conjugated monoclonal antibodies. All staining procedures were carried out at 4 °C in the dark unless otherwise specified. Unstained controls, LIVE/DEAD-only controls, and fluorescent-minus-one controls were included to assess background fluorescence and set gating thresholds. After staining, cells were washed and fixed for 20 minutes using the eBioscience Foxp3/transcription factor fixation buffer set. Fixed samples were stored in the dark at 4 °C until acquisition.

For compensation, single-color controls were prepared using UltraComp eBeads Compensation Beads. Prior to analysis, cells were washed and resuspended, and data were acquired on 100,000 to 500,000 live events per sample using an Aurora flow cytometer (Cytek Biosciences). Flow cytometric data were analyzed with FlowJo software (version 10.8.1; Tree Star).

### Fluorescein Isothiocyanate–Dextran Intestinal Permeability Assay

Intestinal permeability was measured using FITC-dextran (4 kDa). FITC-dextran was administered by oral gavage at a dose of 440 mg/kg body weight (dissolved in sterile PBS). After 4 hours, mice were anaesthetized via intraperitoneal injection of ketamine (100 mg/kg) and medetomidine (1 mg/kg). Mice were positioned in dorsal recumbency once pedal reflexes stopped. Blood was collected from the right ventricle using a closed approach, inserting a 25G needle to the skin until free aspiration into a 1 mL syringe was achieved. Immediately after blood collection, mice were euthanized by cervical dislocation. The blood was collected in serum separator tubes, was left to clot at room temperature for 1 hour before centrifugation at 20,000 g for 3 minutes. Serum FITC-dextran concentrations were measured in duplicate using a fluorescence plate reader (excitation, 485 nm; emission, 528 nm) against a standard curve.

### Quantitative Reverse Transcription Polymerase Chain Reaction

A 0.5-cm segment of distal colon was collected and incubated in RNAlater for 24 hours at 4 °C. Total RNA was extracted using the Qiagen RNeasy kit following the manufacturer’s instructions and was DNase-treated using RQ1 RNase-Free DNase for 30 minutes at 23 °C. RNA purity and quality was measured by NanoDrop 2000 spectrophotometer (ThermoFisher Scientific). Reverse transcription was performed with 2 μg of purified RNA using the High-Capacity cDNA Reverse Transcription Kit. Quantitative reverse transcription polymerase chain reaction was conducted on a StepOnePlus Real-Time PCR (Applied Biosystems) system using SsoAdvanced Universal SYBR Green Supermix. Reactions were run in duplicate, including negative control without complementary DNA template. Results were analyzed using the StepOne software (Applied Biosystems). Gene expression was quantified by the ΔCT (CT is threshold cycle) method relative to the housekeeping gene *Gapdh.* Primer sequences are available in the “List of Primers” file on Figshare https://doi.org/10.6084/m9.figshare.30601727.

### Statistical Analysis and Data Plotting

Sample size was not predetermined using statistical methods. Experimental design followed a randomized block structure, typically including 3 to 5 animals per group per experiment, with each experiment independently replicated at least twice. Data from all animals across replicates were combined for analysis.

Flow cytometry data were analyzed using FlowJo software (v10.8.1; Tree Star). For comparisons between 2 normally distributed groups, statistical significance was assessed using the 2-tailed unpaired *t* test; when normality was not achieved, a nonparametric Mann–Whitney test was performed. When comparing more than 2 groups, analysis of variance was applied. When data was not normally distributed (based on Shapiro–Wilk or Kolmogorov–Smirnov normality tests), a nonparametric test was applied (Kruskal–Wallis test followed by Dunn’s test). To account for multiple comparisons, a false discovery rate threshold of 5% (Q = 0.05) was used, employing indicated post-test correction as implemented in GraphPad Prism version 10.4.0. *P* values adjusted using a false discovery rate that exceeded .05 were considered not significant. All data visualization and statistic analyses were carried out using GraphPad Prism (version 10.4.0; GraphPad Software Inc). Specific statistical parameters for each experiment are provided in the corresponding figure legends.
